# Video-tracked *Anopheles arabiensis* entry and exit behaviour at washed and damaged pyrethroid-treated bednets

**DOI:** 10.1098/rsos.231165

**Published:** 2024-05-29

**Authors:** J. E. A. Parker, C. Kakilla, K. Nelwin, C. Kroner, R. Logan, H. M. Ismail, C. Towers, A. Manjurano, D. Towers, P. J. McCall

**Affiliations:** ^1^ Department of Vector Biology, Liverpool School of Tropical Medicine, Liverpool, UK; ^2^ National Institute of Medical Research, Mwanza, Tanzania; ^3^ School of Engineering, University of Warwick, Coventry, UK; ^4^ Parasitology Department, Heidelberg University Hospital, Heidelberg, Germany

**Keywords:** mosquito, malaria, insecticide-treated bednet, vector control, video tracking, behaviour

## Abstract

Insecticide-treated nets (ITNs) are the most effective method for malaria prevention in Africa. Using near-infrared video tracking in a laboratory environment, we recorded and assessed bednet entry and exit by a northern Tanzanian population of *Anopheles arabiensis* at a human-occupied untreated net and a PermaNet® 2.0 ITN. Both had 12 holes, each 10 cm in diameter, punctured at specific locations, and the ITN was washed 20 times to further simulate the wear and tear of ageing. Washing reduced the insecticide content of ITNs by 61%, which then showed similar rates to the untreated nets for net entry (39% entered untreated net and 41% entered ITN; *p* = 0.84) and exit (37% and 43%, respectively; *p =* 0.67). Regardless of treatment, approximately 40% of mosquitoes entered nets within 20 s of first appearing in the field of view and reached the volunteer’s skin within 5 s of entering the net. Mortality rates post-exposure were significantly higher (*p =* 0.048) at ITNs (26.6%; 95% CI 13.4%–39.7%) than at untreated controls (6.4%; 95% CI 1.8%–14.6%). The washed and aged ITN provided little additional personal protection for the sleeper over an untreated net. Simple adjustments to materials and design that could extend the effective lifespan of ITNs are discussed.

## 1. Introduction

Insecticide-treated nets (ITNs) are the most effective method of malaria prevention and control available for use in Africa [1]. Central to the reductions in malaria transmission sustained over 15 successive years to 2015, they remain the primary method of choice for malaria control even though their efficacy has been diminished by the emergence of pyrethroid resistance, which has already impacted ITN performance in Africa [2]. The threat of resistance to pyrethroids, the insecticide class used by all ITNs to date, has dominated ITN research, driving efforts to identify novel chemical treatments for bednets that would be effective where pyrethroid resistance occurs at a high level [3].

However, even with effective insecticidal treatment, today’s ITNs are far from ideal, and resistance to insecticide is not the only factor compromising performance. Ideally, ITNs are excellent vector control tools with two modes of action: the pyrethroid treatment on the net fibres induces excito-repellency or causes paralysis, eventually leading to mosquito knockdown and death, while the intact net provides a physical barrier that prevents mosquito entry and access to the host. The durability of any particular net refers to its ability to retain sufficient physical integrity and insecticide load to provide such protection for at least 3 years [3].

The poor durability of mass-produced ITNs has been a concern for as long as ITNs have been manufactured commercially, and the view that ITN quality has continued to deteriorate in recent years, despite repeated concerns, is now widespread; many commercial mass-produced ITNs are of questionable quality, i.e. they barely achieve or even fail to satisfy World Health Organization (WHO) standards as measured by wash resistance of the insecticide treatment and durability of the net fibre [3–6].

When pyrethroid resistance appeared in African malaria vectors, attention focused on the identification of novel chemicals for use as effective ITN treatments to replace the existing nets across Africa, thus averting a ‘doomsday’ scenario likely to result from the loss of efficacy of the only treatment available for use with ITNs [3].

Little attention was paid to net durability or the quality of the net fibre, which does not appear to have improved much after decades of use [7]. Affordable bednets are intentionally made of thinner fabrics because heavier nets with thicker fibres create undesirably hot conditions in the poorly ventilated interiors of traditional houses. Hence, when it comes to net quality, there is a necessary trade-off between net strength and net weight, resulting in lighter-weight nets in which the early appearance of holes has had to be tolerated.

There are multiple questions about how insecticidal net treatments might affect a mosquito’s ability to enter a damaged or aged ITN, how they bloodfeed after entering, and how they eventually manage to exit and their fate thereafter. Recent reports estimate that an average of 68% of households in endemic areas of sub-Saharan Africa own at least one ITN [8], but how many of those nets that are still in use have retained their physical integrity and/or insecticidal load to prevent bloodfeeding or to be considered fit for purpose is not known. We report here on a study using video tracking to explore whether the damage typically seen in older nets increases vulnerability to mosquito entry, as measured using a PermaNet® 2.0 bednet (Vestergaard, Lausanne, Switzerland), a first-generation deltamethrin-treated standard ITN, still widely in use across Africa today.

## 2. Material and methods

### 2.1. Mosquitoes

Wild mosquito larvae were collected in the agricultural area of Nsola Village, Magu, Mwanza District, Tanzania (2°29'57.6"S, 33°28'05.2"E), and reared to adults in National Institute for Medical Research (NIMR) insectaries in Mwanza, at 28°C (±4), 80% (±10) humidity, and a 12:12 light:dark period, synchronized with local hours of sunrise and sunset. Adult mosquitoes were fed *ad libitum* on 10% glucose solution and used in testing at 3–5 days post-emergence.

Prior to testing, individual mosquitoes were identified morphologically within a clear plastic aspirator, using the key of Gillies and Coetzee [9]. Only female mosquitoes from the *Anopheles gambiae* species complex were used. These were eventually identified as *A. arabiensis* by the PCR analysis [10] of mosquitoes recaptured after test completion.

#### 2.1.1. Image capture, recording and analysis

Capture, recording and analysis of all images were made using the equipment previously described in detail [11–13]. The main components of this system, which used paired Fresnel lenses on either side of the bed and bednet, were as follows: two Baumer HXC40NIR with near-infrared sensitivity, 2048 × 2048 pixel resolution (Lambda Photometrics, UK); 12.5 mm imaging lens (F1.4, Kowa LM12HC; Multipix, UK); high-power infrared LED (850 nm, Thorlabs, UK); Fresnel lenses (1400 × 1050 mm, NTKJ Co., Japan); personal computer (i7, 3.4 GHz, 8 GB RAM, 20 TB storage; Lambda Photometrics) and StreamPix 5 (Norpix, Canada), all were recorded as uncompressed .seq files, at 50 frames per second and a colour depth of 8 bit greyscale.

Video recordings were analysed by sequential image subtraction to identify mosquito positions and tracking algorithms to connect the mosquito positions into trajectories (the algorithms are fully described in [13]). In turn, each position within a trajectory is classified into one of the four behavioural modes (swooping, visiting, bouncing and resting) using the algorithms originally defined in [11].

#### 2.1.2. Bioassay procedure

**Figure 1.**
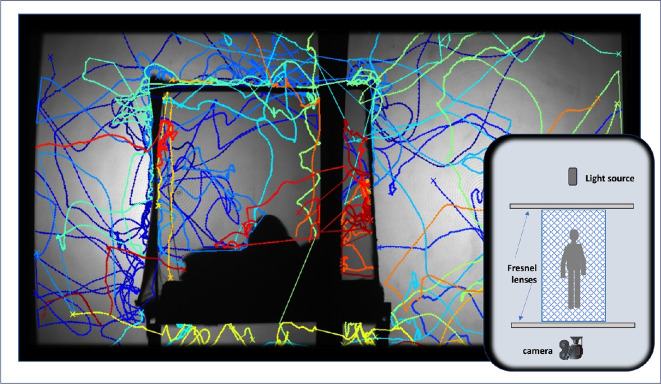
Example of tracked flights of five mosquitoes outside and inside a human-baited bednet. The image shows the broad spatial region occupied by the mosquitoes and the activity of mosquitoes on the outside net surface (pale blue tracks on the roof and top left side) and inside the net (red tracks). The volunteer’s head is visible left of the centre, and the dark vertical line in the centre of the image shows the division between the images from the two cameras. Track line colours illustrate the time of activity (blue = early in the test and red = late in the test). The inset figure shows how the net fitted between the Fresnel lenses, ensuring that all mosquitoes seen framed within the head or foot end of the net were actually inside the net.

All bioassays were conducted in a dedicated test room at the NIMR, Mwanza, Tanzania. The tracking system was operated from a computer located outside the bioassay room (area 6 m× 4 m and height 3 m), within which mosquitoes were allowed to fly freely. A metal-framed single bed was placed in the centre of the room, with a bednet suspended in position supported by an internal frame of carbon fibre rods (4D Modelshop, UK). The smaller head and foot sides of the bednet and bed were positioned to abut with the Fresnel lenses, a constraint imposed by both the bed length and the tracking system’s 2 m maximum depth of field. This setup proved advantageous because it prevented mosquito flight outside the Fresnel lenses. Hence, all activity perceived within the net when viewed through the short head and foot ends of the net could only have occurred inside the net (see figure 1*b*).

#### 2.1.3. Bednets

We tested the PermaNet 2.0 bednet after 20 washes and with 12 holes cut to simulate wear and tear [14] and tested an untreated polyester net (Coghlan’s, USA) as a control. Nets were hand washed according to the procedures described in WHO guidelines [14] with some adjustments according to local resource availability. As Savon de Marseille was unavailable at the location, Palmer’s Olive Oil Butter was substituted as an equivalent fragrance-free olive oil-based soap (Palmer’s, USA). Water was not tested for hardness. Nets were line-dried outdoors, with 1 day’s regeneration time between each wash.

Following use in the test, the insecticide residue on the nets was quantified as previously described [15] with some modifications. Briefly, an area of 8.04 cm^2^ net sample was cut from each net panel in triplicate for treated nets and only one replicate on untreated nets. The active ingredient was extracted from the net samples by heating with an extraction solution containing 10% 1-propanol in heptane containing 100 μg dicyclohexyl phthalate (DCP) as an internal standard at 85°C for 45 min. After heating the samples, 1 ml of the extraction solution was removed and evaporated under nitrogen. The residue was resuspended in 1 ml of acetonitrile, and samples were filtered through Polytetrafluoroethylene (PTFE) 0.2 m filters with 17 mm diameter. A Hypersil Gold C18 Reverse-Phase column was used at 21°C to separate and analyse 10 μl of the extract on an Agilent 1100 series High Performance Liquid Chromatography (HPLC). The active ingredient was detected and quantified using an isocratic separation condition using 70% acetonitrile and 30% water with a flow rate of 1 ml/min and an absorbance wavelength of 226 nm. A standard curve of authentic deltamethrin standards was used to quantify deltamethrin concentrations detected in treated net extracts [15].

**Figure 2.**
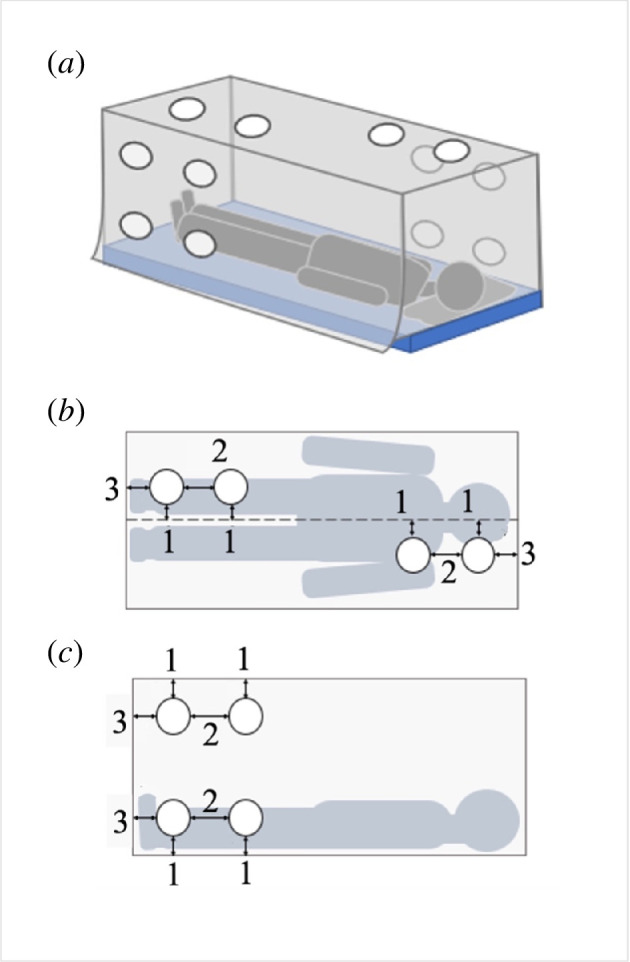
Location of holes introduced into test bednets. (*a*) Schematic showing the location of twelve 10 cm holes in the bednet, and the untucked edge on one long side of the net. (*b*) Positions of holes on the bednet roof. Holes on the roof were displaced 4 cm from the roof centre (arrow 1), with a 15 cm gap between hole pairs (arrow 2). The holes were 10 cm away from the edge of the net (arrow 3). The dotted line indicates the centre of the net. (*c*) Positions of holes on the bednet side walls. Holes on the side of the net were positioned 10 cm from the top/ bottom edges of the net (arrows 1 and 3) with a 20 cm gap between hole pairs (arrow 2).

A total of 12 circular holes, 10 cm in diameter, were cut at six locations in each net, as shown in figure 2*a*. When the net was fitted, one side was intentionally left untucked at the base, leaving an open gap of 2 cm between the bottom of the net and the bed. The other three sides were tucked under the mattress. The exact positions of the holes are shown in figure 2*c,d*. By limiting the number of holes on opposite sides of the net, their position relative to the volunteer was known. For example, in figure 2*a*, holes on the left side of the net are adjacent to the volunteer’s feet, while holes on the right side of the net are adjacent to the volunteer’s head. When viewed through the camera (figure 2*b*) holes on the left side of the net were referred to as being at the ‘foot end’ of the net, and those on the right side were at the ‘head end’.

Hole locations were chosen to explore the relative hazards of net damage at different heights from the ground and in different locations relative to the volunteer’s body. Since mosquitoes show preferences for different net surfaces [11], it was expected that holes in ‘high contact’ net locations would admit more mosquitoes than those in less-preferred areas. The untucked net side was included to investigate the risk posed by a loose hanging net.

#### 2.1.4. Test procedure

**Figure 3.**
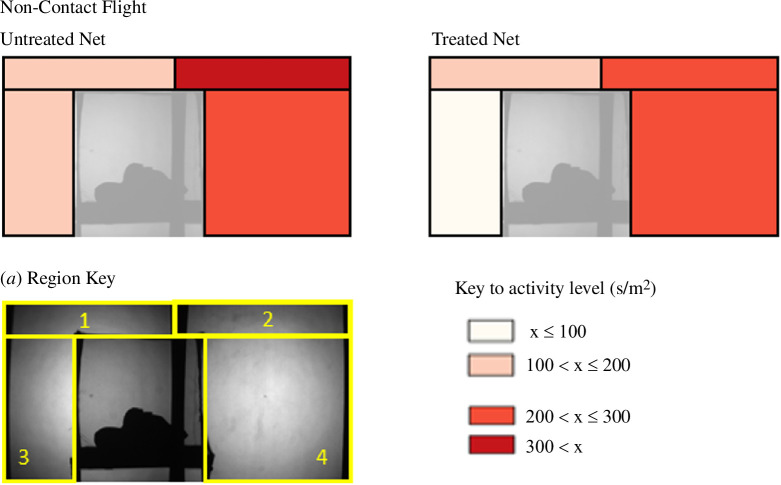
Variation in swooping flight activity in different regions around the bednet. Swooping flights make no contact with the bednet surface. To present flight activity in a way that is comparable between spatial regions of markedly different sizes, flight activity is presented here as seconds per square metre. Regions represent different airspaces above (regions 1 and 2) and adjacent to (regions 3 and 4) the bednet. Since this recording was made in two dimensions, airspace values are given in square metres.

Each test used five female mosquitoes, sugar starved for 11 hours prior to testing. At 1 hour before the test, the cup containing the mosquitoes was transferred to the room, placed at eave height. Thirty minutes later, half an hour prior to the test, the volunteer entered the room and lay under the net. The mosquito release position was located at the upper right-hand of zone 2, as shown in figure 3*a*, outside the field of view of cameras.

To start the test, the observer stationed outside the room pulled a string to remove the net cover and inverted the paper cup, releasing mosquitoes. Mosquitoes were allowed to fly and seek hosts unhindered and unaided within the room for 2 hours, and all flight activities around the ITN were recorded on video.

At the end of the test period, mosquitoes were collected using a battery-powered aspirator (Spider Vacuum, SK Depots, UK), recording where they were collected (inside or outside of the net) and whether they had blood-fed. Mosquitoes were then provided with 10% sucrose solution on a cotton pad and held for 24 hours to assess post-test mortality.

#### 2.1.5. Data analysis

With a few exceptions, all data were analysed using linear mixed models (lme4 in R [16]), using explanatory variables of net treatment, and the sex of the volunteer. Volunteer identity was included as a random effect.

A few variables were analysed using other methods, as described in the following sections.

##### 2.1.5.1. Activity by net region

For the analysis of the activity in different spatial regions of the net, data were scaled to account for the differing sizes of the regions and analysed using linear mixed models (lme4 package in R [16]). Volunteer identity was used as a random effect, and the net type and area name were input as explanatory variables.

##### 2.1.5.2. Net entry, exit, bloodfeeding and mortality

Proportion data (proportion entering, exiting, bloodfeeding and mortality) were analysed in SAS Studio University Edition (SAS, USA) using generalized estimating equations (glimmix package). Volunteer identity was included as a random effect. The exit rate was scored as the proportion of mosquitoes that entered the net and managed to exit.

Entry location and timing were assessed by manual examination of tracks produced by the tracking software. The use of small mosquito groups allowed accurate assessment of entry time in most entry tracks (69 of 72 cases) and all exit tracks (31 of 31 cases). The location of net transit was ambiguous in three entry tracks (all exit tracks were clearly visible). If cases were ambiguous, the data points were excluded from the analyses of entry timing and location trends.

##### 2.1.5.3. Net contact ‘per mosquito’

Contact with the net was analysed in its original form (i.e. total contact accrued by all five mosquitoes throughout the 2 hour recording). For ease of interpretation, it was also presented in ‘per mosquito’ format with two values calculated differently: the first assumed all five mosquitoes responded to the host and made equal contact with the net, i.e. total contact divided by 5 to generate a value of contact per mosquito. The second recognized that not all of the five mosquitoes released will have approached and contacted the net equally and used the maximum number seen together in view at any one time (i.e. the number known to have responded) and divided the total contact by this value. For example, in the latter metric, if only three mosquitoes were viewed in the frame at the same time, the higher contact time value would be calculated by dividing the total contact time by 3.

### 2.2. Ethical considerations

All participants who volunteered to act as sleepers within the nets were recruited from the local population. All were adults (9 women and 10 men) and all provided written informed consent before participation. Seventeen volunteers took part in two tests (one with a treated net and another with an untreated net). In the case of the 18th test, two different volunteers were used for treated/untreated owing to volunteer dropout. NIMR policy does not automatically insist on the provision of anti-malarial prophylaxis for volunteers. Instead, all volunteers sleeping under ITNs were monitored daily for signs of fever for 2 weeks post-exposure. If sickness was detected, the individual was referred to a nearby health facility with the cost of treatment borne by the project.

## 3. Results

### 3.1. Identification of mosquito species

A subsample of 100 female mosquitoes used in the assays was tested by PCR to determine *A. gambiae* species complex identity. All but one were identified as *A. arabiensis*. The remaining mosquito was PCR negative and considered to be a species outside the *A. gambiae* species complex.

### 3.2. Insecticide content of washed nets

HPLC tests showed that PermaNet 2.0 washed 20 times to simulate ageing contained 21.5 mg/m^2^ deltamethrin (s.d. ±3.7) and confirmed that untreated control nets had 0 mg/m^2^ (s.d. ±0). For PermaNet 2.0, this represented a 61% decrease from the advertised pre-washing insecticide content of 55 mg/m^2^.

### 3.3. Mosquito entry and exit rates

Figure 1 includes examples of mosquito flight tracks approaching, entering, moving outside and inside and exiting a human-baited bednet.

Of the 36 tests conducted, at least one mosquito entered the net in 29 tests; entries were observed in 15 treated net tests and 14 untreated net tests; a total of 37 mosquito entries were counted with untreated nets and 35 in washed PermaNet 2.0. Entry success rates did not differ significantly between treated and untreated nets; a mean of 39% (95% CI: 25%–53%) of mosquitoes successfully entered an untreated net, compared with 41% (95% CI: 23%–59%) at a treated net (generalized estimating equation (GEE) test statistic: *F*_1, 16_ = 0.04, *p* = 0.84). That is, of the five mosquitoes released, an average of two gained entry to the holed nets within the 2 hour period of a test.

Insecticide treatment did not significantly affect mosquito exit success (GEE: *F*_1, 10_ = 0.19, *p =* 0.67). A mean of 37% (95% CI: 16%–58%, *n* = 14) of mosquitoes that entered the untreated net exited the net before the end of the 2 hour test compared with 43% (95% CI: 21%–65%, *n* = 5) of mosquitoes at a treated net.

### 3.4. Mosquito bloodfeeding rate

Bloodfeeding rate, the proportion of bloodfed mosquitoes among those recaptured at the end of each test, was not significantly affected by net treatment (GEE: *F*_1, 16_ = 2.06, *p =* 0.17) as 37% (95% CI: 20%–55%) of mosquitoes fed in the untreated group compared with 24% (95% CI: 13%–34%) in the treated net tests.

### 3.5. Mosquito flight

**Figure 4.**
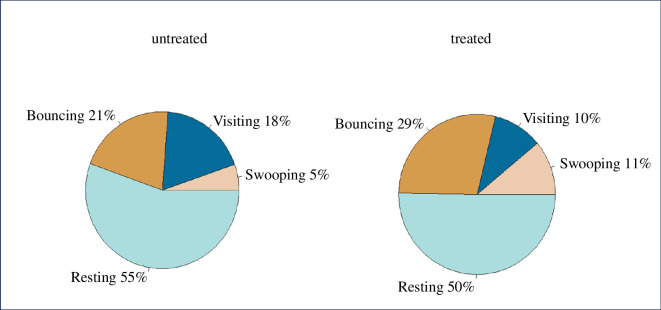
Activity by (mean) behaviour mode at untreated and treated nets. There were no significant differences in any behaviour mode in treated versus untreated nets, as tested by linear mixed models (swooping LMM: *F*_1,19_ = 0.77, *p* = 0.39; visiting LMM: *F*_1,18_ = 3.11, *p* = 0.10; bouncing LMM: *F*_1,18_ = 0.04, *p* =0.85; resting LMM: *F*_1,18_ = 3.12, *p* = 0.09). Behaviour modes, as defined in Parker *et al*. (2015), describe tracks with these characteristics: swooping: no contact with the bednet; visiting: relatively long periods of flight interspersed with infrequent net contacts; bouncing: multiple rapid contacts with the bednet surface at intervals of less than 0.4 s; resting: mosquito was either completely static for at least 0.75 s or where the movement velocity was less than 1.33 mm/s.

Mosquito activity was visible in the field of view for significantly longer time periods in untreated net tests compared with treated net tests (LMM: *F*_1, 17_ = 4.79, *p =* 0.04; untreated net activity, 52.9 min (95% CI: 29.3%–76.6%); treated net activity, 35.4 min (95% CI: 14.7%–56.2%)). However, this difference in activity level was not the result of any shift in behaviour modes (defined in [11]). No particular mode was affected more than others, and the proportion of time in all four modes was similar with both treated and untreated nets (figure 4).

Non-contact flight (‘swooping’ behaviour mode) in the air space around the bednet (figure 3) was evenly distributed in all areas, with no significant effect of area (LMM: *F*_3, 120_ = 2.02, *p =* 0.12) or net treatment (LMM: *F*_1, 123_ = 0.07, *p =* 0.79) on flight activity.

**Figure 5.**
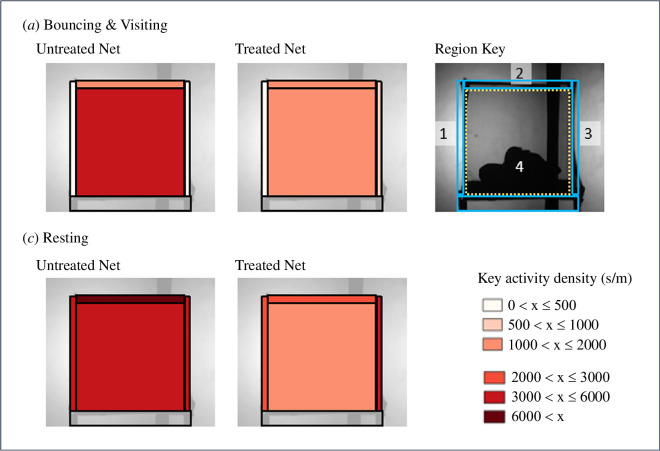
Variation in contact flight activity at different regions/surfaces on the bednet. Illustrating the range of bouncing, visiting and resting times recorded for mosquitoes on the different net surfaces. Note that contact in region 4 here includes mosquitoes that contacted the human volunteer, thus a high proportion of time spent at this region would not involve contact with the fabric of the net.

Flights involving brief contact with the net (‘visiting’ and ‘bouncing’ behaviour modes) were unevenly distributed across the net with higher levels observed on the roof of the net (LMM: *F*_3, 119_ = 6.77, *p =* 0.0003) though net treatment did not significantly impact the time mosquitoes spent engaged in these flight modes (LMM: *F*_3, 122_ = 0.49, *p =* 0.48). As shown in figure 5, higher levels of these ‘brief contact’ flights were recorded within the net (region 4), where flights would have involved contact with both the net and the volunteer’s body.

Resting flight was roughly evenly distributed across different areas (LMM: *F*_3, 135_ = 0.28, *p =* 0.84), with no evidence of any change in activity levels resulting from net treatment (LMM: *F*_1, 135_ = 0.65, *p =* 0.42, figure 5).

### 3.6. Net contact

Net contact values calculated for the entire group and per individual mosquito are given in table 1. Using conservative calculations to estimate the individual contact made per mosquito, it is estimated that mosquitoes in all treatments contacted the net for over 4 min each (6.7 min (95% CI: 3.9–9.5) untreated nets; 4.2 min (95% CI: 1.6–6.9) treated nets). Net treatment did not significantly affect the duration of net contact (LMM: *F*_1, 18_ = 3.36, *p =* 0.08).

**Table 1.** Mosquito contact times at untreated or treated and washed nets (minutes). The first column ‘total’ represents contact time accrued by all five mosquitoes over the course of the 2 hour filming period. The last two columns represent ‘per mosquito’ contact estimates, calculated using different assumptions about the proportion of the five mosquitoes that responded to the baited net.

washed net	total (min)	contact per mosquito (min)	contact per mosquito (max)
**untreated**	33.5 (19.3–47.7)	6.7 (3.9–9.5)	14.1 (6.8–21.4)
**treated**	21.1 (7.9–34.3)	4.2 (1.6–6.9)	8.0 (2.4–13.5)

### 3.7. Entry locations

**Figure 6.**
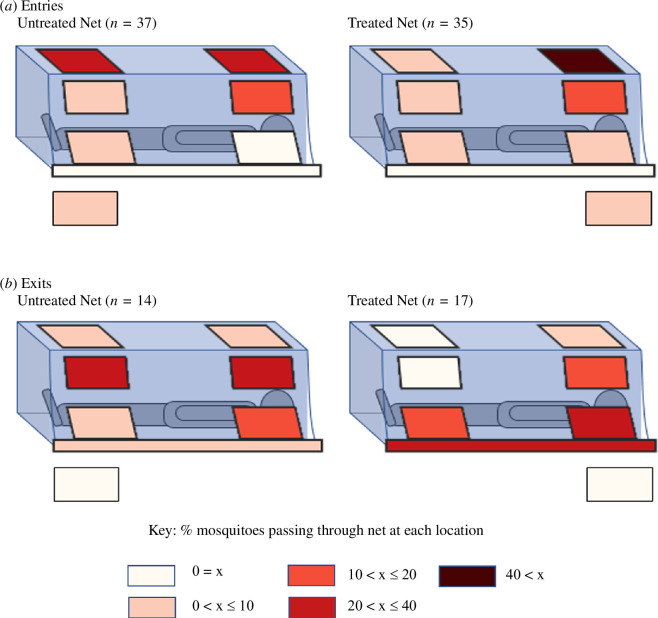
(*a*) Entry and (*b*) exit locations of mosquitoes at holed bednets. The coloured rectangles mark the position of each pair of 10 cm circular holes. The rectangle beneath each net represents instances when the entry or exit location was ambiguous or indeterminate. One edge of the net was left untucked. Values shown are the percentage of all entries/exits that occurred in each location.

Entry locations were unevenly distributed across the bednet (LMM: *F*_7, 256_ = 12.36, *p >* 0.0001), as significantly more entries occurred through the hole in the roof, above the volunteer’s head (*t* = 6.35, *p >* 0.0001; figure 6). There was a significant interaction between net treatment and entry location (LMM: *F*_7, 256_ = 3.30, *p =* 0.002), with more entries at the foot end of the roof of the untreated net (*t* = 2.50, *p =* 0.01) and slightly fewer entries at the head end (*t* = −2.0, *p =* 0.0468; figure 6*a*).

### 3.8. Exit locations

Exit locations (figure 6*b*) were unevenly distributed across the bednet (LMM: *F*_7, 256_ = 2.17, *p =* 0.036), with most mosquitoes exiting via the net’s sides at the sleeper’s upper body. The exit route was not influenced by net treatment (LMM: *F*_1, 263_ = 0.20, *p =* 0.65).

### 3.9. Knockdown and mortality

Net treatment had a significant impact on knockdown following tests (GEE: *F*_1, 16_ = 6.08, *p =* 0.03), with 19.7% (95% CI: 7.3%–32%) of mosquitoes found knocked down at the end of tests with treated nets compared with 3.6% (95% CI: −0.6% to 7.8%) in untreated net tests. At 24 hour post-exposure, net treatment continued to show a significant impact on mortality with 26.6% mortality (95% CI: 13.4%–39.7%) compared with 6.4% with untreated nets (95% CI: −1.8% to 14.6%; GEE: *F*_1, 16_ = 4.58, *p =* 0.048).

### 3.10. Mosquito behaviour

In total, 35 mosquitoes successfully entered holes in treated nets and 37 entered untreated nets. The tracks of these flights were examined in further detail.

### 3.11. Time of appearance

On average, the mosquitoes that successfully entered the net did so less than an hour after they were released into the bioassay room (untreated nets: 35.1 min (28.3–41.9), *n* = 35; treated nets: 34.7 min (27.7–41.8, *n* = 34); means (95% CI), *n* = 36). Net treatment did not affect the time taken to entry (LMM: *F*_1, 16_ = 0.01, *p =* 0.93).

### 3.12. Time from appearance to entry

On average, mosquitoes took less than 20 s from first appearing in the field of view to successfully locating and entering through a hole in the net (delay from appearance to entry; 19.5 s (15.8–23.3) in untreated nets; 18.0 s (14.0–22.1) in treated nets; mean (95% CI), *n* = 35, 34, respectively; three mosquitoes’ entry time could not be accurately ascertained). Net treatment did not significantly influence entry time (LMM: *F*_1, 65_ = 0.26, *p =* 0.61).

### 3.13. Entry to ‘touchdown’ on a human host

The mean time from entry to apparent ‘touchdown’ on the human was just under 5 s. This was unaffected by net type (untreated net: mean 4.9 s (3.8–6.0), *n* = 33; treated net: mean 4.9 s (3.7–6.2), *n* = 33; means (95% CI); LMM: *F*_1, 57_ = 0.01, *p =* 0.92).

### 3.14. Exitingthe net

A total of 17 mosquitoes exited the treated nets compared with 14 exiting the untreated nets. The duration of time spent inside the net before exit was not significantly affected by net treatment (LMM: *F*_1,28_ = 0.00, *p =* 0.98): mosquitoes spent 32.2 min (95% CI: 20.1–44.3) with untreated nets compared with 28.6 min (95% CI: 14.8–42.4) with treated nets.

The first mosquitoes entering the nets did so approximately 35 min after release and then did so with only brief contact prior to entry. After entry, mosquitoes stayed inside for 28–36 min before exiting the net. Even without directly measuring contact, clearly, the majority of net contact by mosquitoes that entered the net was made with the interior of the net and after they had landed on the volunteer.

## 4. Discussion

The level of dependency on a simple physical device like an ITN, as the primary, or the only, method available for communities to protect families from a fatal infection, is exceptional. Considering how important ITNs had been in reducing malaria cases prior to 2015, the knowledge that today, many new nets are barely fit for purpose and that after only a couple of years of use, most no longer provide adequate protection to those using them [3–6], is a sobering thought and demonstrates our tolerance of a level of quality so low that it must surely be on the borderline of acceptability.

ITNs acquire holes rapidly in routine daily use. In Zambia [17], 94% of nets had holes after 2.5 years of daily use, while in Uganda, the proportion of nets classified as ‘too torn’ on the WHO’s Proportionate Hole Index (pHI) scale was 0.066, with this proportion approximately doubling after 25 months to 0.125 [6]. In another Zambian study, of 33% of nets that were still in use after 30 months, only half met the criteria for functional survival [18]. The 10 cm diameter hole sizes used in the present study were larger than the standard hole size for WHO testing, but at 78.5 cm^2^ in diameter, they were within the ranges found in field settings. Vanden Eng *et al.*’s study of used nets in Malawi reported median hole sizes of 13 cm^2^, with quartile ranges from 3 to 101 cm^2^ (first quartile, third quartile) [19]. The level of artificial damage added to the nets in the current bioassay was within the bounds of what can be found in the field, though typically large holes in nets are long and narrow, rather than circular [1]. Nets with the damage levels used in the present study (785 cm^2^ total hole area) would fall into the ‘damaged’ category if classified by the pHI (category ‘damaged’ = 80–789cm^2^ total hole area; category ‘too torn’ = over 790 cm^2^ total hole area) [18]. This integrity category represents nets with reduced efficacy, though they provide more protection than no net at all.

Over time, ITNs also lose insecticidal efficacy [19–21], as nets gather deposits of dust, sweat and sebum, especially when washed repeatedly. Notably, the PermaNet 2.0 is reported to perform very poorly in this respect, retaining only 18%–27% of the initial insecticide content after 20 washes [19], a slightly greater loss in efficacy than the 39% insecticide retention after 20 washes recorded in the present study. Newer net designs incorporate the insecticide within the fibres of the material rather than simply coating the fibres with superficial layers of insecticide (the method used for the PermaNet 2.0). Recent studies report few differences in behavioural responses in *A. gambiae s.l.* following exposure to a range of nets with different active ingredients regardless of how they were originally loaded with active ingredients [9]. However, while there may be no difference in performance when both nets are new, the rapid rate at which insecticidal efficacy is lost by a coated fibre compared with incorporated insecticides means that the latter type of net should still be providing adequate protection long after the older net is no longer protective.

Evaluating how the aged PermaNet 2.0 performed in comparison with untreated nets, we found no significant differences in the entry or exit rates or bloodfeeding rates between the two bednet types. Thus, regardless of net treatment, approximately 40% of mosquitoes entered nets approximately 35 min after release, of which 24% and 37% engorged on blood in treated and untreated nets, respectively; eventually, approximately 40% of those that entered either bednet type escaped from it. With no significant difference between any of these key measures, the results demonstrate no significant personal protection benefits for the user of an aged PermaNet 2.0 over a user of an untreated net. Comparison with the performance of a new unwashed PermaNet 2.0 in a previous study [11] shows great differences in the behavioural modes to the extent that the washed net in the present study resembles the untreated control more than the ITN. As such, a holed new net would be expected to offer better protection than the artificially aged net used in this study.

In our tests, mosquitoes located the human sleeper rapidly on arrival at the net. On an average flight trajectory, less than 20 s elapsed from the moment the mosquito first appeared in the cameras’ field of view to when it located and entered the hole successfully. Mosquitoes entering the net would fly directly to the host to feed, on average, within 5 s of entering the net. This behaviour would enable the mosquito to avoid contact with the treated net until after bloodfeeding when the bloodmeal confers a significant level of resistance on the otherwise susceptible mosquito [22,23].

*Anopheles gambiae s.l.* appear to be well-equipped to respond to or evade insecticides on bednets. Fatou and Müller [24] found that while permethrin-treated nets had no detectable repellent effect on mosquitoes, they elicited a strong irritant response resulting in contact disengagement, allowing both susceptible and resistant forms to pass more easily through holes in treated nets than untreated nets. Barreaux *et al*. [25] recently reported that although the duration of bloodfeeding was significantly reduced when feeding through an ITN, *A. gambiae s.l.* could compensate by increasing its ingested blood flow rate by 35%.

Although this study was confined to pyrethroid-susceptible mosquitoes, many findings are equally applicable to next-generation ITNs used against resistant populations of *A. gambiae s.l.* in Africa. Aged nets have lower insecticide content and will impact less on the target population. Mechan *et al*. [6] found that two next-generation ITNs, Olyset Plus and PermaNet 3.0, were effective against a pyrethroid-resistant strain at baseline, but bio-efficacy fell over time until they reached a point after 2 years of operational use, when mortality rates fell to 26% and 46%, respectively. Lukole *et al*. [26] reported that after 3 years in use against resistant populations, both the PermaNet 2.0 and the Piperonyl Butoxide (PBO) net PermaNet 3 retained similar levels of deltamethrin. The rate of loss of bio-efficacy was slower in PermaNet 3, which, regardless of condition, remained more protective than the standard PermaNet 2.0 nets after 3 years of use.

Which of the two consequences of ageing is more important for vector control: the increase in physical damage or the loss of insecticide? The negative consequences of holes in nets may impact earlier. Targeting a moderately resistant vector population in Malawi, Shah *et al.* [27] found that 1 or 2 years of old ITNs did not impact the incidence of malaria in children, but among ITN users, there was an increase in protection provided by ITNs without holes compared with ITNs with holes.

In our tests with PermaNet 2.0 bednets reported here, the majority of mosquitoes entered the net through the holes in the roof. This is not surprising, given that most mosquito activity occurs above the roof and the majority of mosquito contacts with the net occur on the roof [11–13]. Sutcliffe and Colborn [28] reported that the chances of a mosquito finding a hole in the roof of an untreated net were higher than on the side of the net, with a 20% higher chance of entering it. Considering the exceptionally high proportion of mosquito activity that occurs on or above the bednet roof, this would be a worrying report, were it not for the fact that holes in the bednet roof are very rare. Field studies consistently report few or no holes on the top of ITNs in regular use; by contrast, the highest number of holes found in bednets typically are in the lowest quarter of each net side [6,17,29]. The lack of coincidence between vector entry preference and location vulnerable to entry is very fortunate from a human point of view. By contrast, mosquitoes preferred to exit nets from holes closer to the ground and through the untucked edge of the net. While advice to net users to tuck nets into beds to secure them remains important, this study would indicate that in heavily damaged nets, an untucked edge is more commonly used for net exit than entry. Using a stronger more durable fibre on the sides of the ITN may prove to be a better feature than ensuring that an insecticide at the sides of the net is delivered as effectively as it is elsewhere on the bednet.

### 4.1. Conclusion

Holes in an ITN roof and upper sides are more easily encountered and entered by the African malaria vector *A. arabiensis* since these areas are more frequently approached and contacted by mosquitoes [11,12]. Fortunately, the region most vulnerable to physical damage and insecticide loss is the lowest part of the sides. Field studies consistently report few or no holes on the top of ITNs in regular use, indicating that the existing net roof requires little alteration as long as it is treated with an effective insecticide that is picked up easily by mosquitoes and that has good prospects for a long lifespan before resistance emerges.

By contrast, the highest number of holes typically found in bednets is in the lowest quarter of each net side [6,27,28]. An immediate solution here would be to reduce the risk of holes developing later, either by strengthening the fibre used on the sides of the net or by using a double layer of netting in these vulnerable areas. This simple adjustment could be made to virtually all ITN brands marketed today.

However, as long as the choice of ITNs for large-scale deployment is determined by cost as much as, or more than, effectiveness or longevity, better ITN designs are unlikely to become reality.

## Data Availability

All raw data supporting this article are included in the manuscript and supplementary information [30]. Video recordings were analysed by sequential image subtraction to identify mosquito positions and tracking algorithms to connect the mosquito positions into trajectories (the algorithms are fully described in [13]). In turn, each position within a trajectory is classified into one of four behavioural modes (swooping, visiting, bouncing and resting) using the algorithms originally defined in [11].

## References

[1] Bhatt S *et al* . 2015 The effect of malaria control on Plasmodium falciparum in Africa between 2000 and 2015. Nature **526** , 207–211. (10.1038/nature15535) 26375008 PMC4820050

[2] Toé KH , Jones CM , N’Fale S , Ismail HM , Dabiré RK , Ranson H . 2014 Increased pyrethroid resistance in malaria vectors and decreased bed net effectiveness, Burkina Faso. Emerg. Infect. Dis. **20** , 1691–1696. (10.3201/eid2010.140619) 25279965 PMC4193182

[3] Lindsay SW , Thomas MB , Kleinschmidt I . 2021 Threats to the effectiveness of insecticide-treated bednets for malaria control: thinking beyond insecticide resistance. Lancet. Glob. Health **9** , e1325–e1331. (10.1016/S2214-109X(21)00216-3) 34216565

[4] Vinit R *et al* . 2020 Decreased bioefficacy of long-lasting insecticidal nets and the resurgence of malaria in Papua New Guinea. Nat. Commun. **11** , 3646. (10.1038/s41467-020-17456-2) 32686679 PMC7371689

[5] Wheldrake A , Guillemois E , Arouni H , Chetty V , Russell SJ . 2021 Textile testing to assess the resistance to damage of long-lasting insecticidal nets for malaria control and prevention. Malar. J. **20** , 1–17. (10.1186/s12936-020-03571-4) 33468152 PMC7816374

[6] Mechan F *et al* . 2022 LLIN evaluation in Uganda project (LLINEUP): The fabric integrity, chemical content and bioefficacy of long-lasting insecticidal nets treated with and without piperonyl butoxide across two years of operational use in Uganda. Curr. Res. Parasitol. Vector. Borne. Dis. **2** , 100092. (10.1016/j.crpvbd.2022.100092) 35734077 PMC9207544

[7] Gleave K , Lissenden N , Chaplin M , Choi L , Ranson H . 2021 Piperonyl butoxide (PBO) combined with pyrethroids in insecticide-treated nets to prevent malaria in Africa. Cochrane Database Syst. Rev. **5** , CD012776. (10.1002/14651858.CD012776.pub3) 34027998 PMC8142305

[8] WHO . 2022 World malaria report 2022. Geneva: World Health Organization.

[9] Gillies MT , Coetzee M . 1987 A supplement to the Anophelinae of the South of the Sahara (Afrotropical region). Publ. South African Inst. Med. Res. 1–143.

[10] Scott JA , Brogdon WG , Collins FH . 1993 Identification of single specimens of the Anopheles gambiae complex by the polymerase chain reaction. Am. J. Trop. Med. Hyg. **49** , 520–529. (10.4269/ajtmh.1993.49.520) 8214283

[11] Parker JEA , Angarita-Jaimes N , Abe M , Towers CE , Towers D , McCall PJ . 2015 Infrared video tracking of Anopheles gambiae at insecticide-treated bed nets reveals rapid decisive impact after brief localised net contact. Sci. Rep. **5** , 13392. (10.1038/srep13392) 26323965 PMC4642575

[12] Parker JEA , Angarita Jaimes NC , Gleave K , Mashauri F , Abe M , Martine J , Towers CE , Towers D , McCall PJ . 2017 Host-seeking activity of a Tanzanian population of Anopheles arabiensis at an insecticide treated bed net. Malar. J. **16** , 270. (10.1186/s12936-017-1909-6) 28676092 PMC5496219

[13] Angarita-Jaimes NC , Parker JEA , Abe M , Mashauri F , Martine J , Towers CE , McCall PJ , Towers DP . 2016 A novel video-tracking system to quantify the behaviour of nocturnal mosquitoes attacking human hosts in the field. J. R. Soc. Interface **13** , 20150974. (10.1098/rsif.2015.0974) 27075002 PMC4874425

[14] World Health Organization . 2013 Guidelines for laboratory and field-testing of long-lasting insecticidal nets [Internet]. WHO/HTM/NTD/WHOPES/2013.1. Geneva, Switzerland. See http://apps.who.int/iris/bitstream/handle/10665/80270/9789241505277_eng.pdf?sequence=1

[15] Walker KJ , Williams CT , Oladepo FO , Lucas J , Malone D , Paine MJI , Ismail HM . 2022 A high-throughput HPLC method for simultaneous quantification of pyrethroid and pyriproxyfen in long-lasting insecticide-treated nets. Sci. Rep. **12** , 9715. (10.1038/s41598-022-13768-z) 35690679 PMC9188574

[16] Bates D , Maechler M , Bolker B , Walker S . 2015 Fitting linear mixed-effects models using “lme4.” J. Stat. Softw **67** , 1–48. (10.18637/jss.v067.i01)

[17] Craig AS *et al* . 2015 Long-lasting insecticidal nets in Zambia: a cross-sectional analysis of net integrity and insecticide content. Malar. J. **14** , 239. (10.1186/s12936-015-0754-8) 26054336 PMC4470130

[18] Vanden Eng JL *et al* . 2017 Assessing bed net damage: comparisons of three measurement methods for estimating the size, shape, and distribution of holes on bed nets. Malar. J. **16** , 405. (10.1186/s12936-017-2049-8) 29017537 PMC5635507

[19] Atieli FK , Munga SO , Ofulla AV , Vulule JM . 2010 Wash durability and optimal drying regimen of four brands of long-lasting insecticide-treated nets after repeated washing under tropical conditions. Malar. J. **9** , 248. (10.1186/1475-2875-9-248) 20799996 PMC2936406

[20] Gimnig JE , Lindblade KA , Mount DL , Atieli FK , Crawford S , Wolkon A , Hawley WA , Dotson EM . 2005 Laboratory wash resistance of long-lasting insecticidal nets. Trop. Med. Int. Health **10** , 1022–1029. (10.1111/j.1365-3156.2005.01481.x) 16185237

[21] Atieli FK , Munga SO , Ofulla AV , Vulule JM . 2010 The effect of repeated washing of long-lasting insecticide-treated nets (LLINs) on the feeding success and survival rates of Anopheles gambiae. Malar. J. **9** , 304. (10.1186/1475-2875-9-304) 21029477 PMC2988039

[22] Spillings BL , Coetzee M , Koekemoer LL , Brooke BD . 2008 The effect of a single blood meal on the phenotypic expression of insecticide resistance in the major malaria vector Anopheles funestus. Malar. J. **7** , 226. (10.1186/1475-2875-7-226) 18973704 PMC2584071

[23] Hughes A , Lissenden N , Viana M , Toé KH , Ranson H . 2020 Anopheles gambiae populations from Burkina Faso show minimal delayed mortality after exposure to insecticide-treated nets. Parasit. Vectors **13** , 17. (10.1186/s13071-019-3872-2) 31924276 PMC6954553

[24] Fatou M , Müller P . 3D video tracking analysis reveals that mosquitoes pass more successfully through holes of a permethrin-treated than an untreated net. See https://www.researchsquare.com/article/rs-3006212/v1 10.1038/s41598-024-63968-y PMC11169678 38866869

[25] Barreaux P , Ranson H , Foster GM , McCall PJ . 2023 Exposure to pyrethroid-treated bed nets impair blood feeding performance in insecticide resistant mosquitoes. Sci. Rep. **13** , 10055. (10.1038/s41598-023-35958-z) 37344580 PMC10284836

[26] Lukole E *et al* . 2022 Protective efficacy of holed and aging PBO-pyrethroid synergist-treated nets on malaria infection prevalence in north-western Tanzania. PLOS Glob. Public Health **2** , e0000453. (10.1371/journal.pgph.0000453) 36962517 PMC10022078

[27] Shah MP *et al* . 2020 The effectiveness of older insecticide-treated bed nets (ITNs) to prevent malaria infection in an area of moderate pyrethroid resistance: results from a cohort study in Malawi. Malar. J. **19** , 24. (10.1186/s12936-020-3106-2) 31941502 PMC6964029

[28] Sutcliffe J , Colborn KL . 2015 Video studies of passage by Anopheles gambiae mosquitoes through holes in a simulated bed net: effects of hole size, hole orientation and net environment. Malar. J. **14** , 199. (10.1186/s12936-015-0713-4) 25962596 PMC4457991

[29] Tan KR *et al* . 2016 A longitudinal study of the durability of long-lasting insecticidal nets in Zambia. Malar. J. **15** , 106. (10.1186/s12936-016-1154-4) 26891696 PMC4759777

[30] Parker JE , Kakiila C , Nelwin K , Kröner C , Logan R , Ismael H . 2024 Data from: Video tracked Anopheles arabiensis entry and exit behaviour at washed and damaged pyrethroid-treated bed nets. Figshare (10.6084/m9.figshare.c.7184005) PMC11285834 39076790

